# Supplementary material for: Characterizing the suckling behavior by video and 3D-accelerometry in humpback whale calves on a breeding ground
Source: PeerJ. 2022 Feb 17;10:e12945. doi: 10.7717/peerj.12945 (PMC8858581; doi:10.7717/peerj.12945)
Supplement: Supplemental Information 1 — The data derived from the 3-axis accelerometer and the depth sensor of the tags, sampled at 10 Hz, was split into 2 s non-overlapping blocks (windows) and each block was labeled either as ‘suckling’ or ‘non-suckling’ depending on which behavioral period it fell under (suckling period or any non-suckling period). The data underwent segmentation in order to partially remove noise and also reduce the class imbalance. The thresholds we used (< 1.5 m depth and > 2 m s–1 speed) were based on the known characteristics of the nursing/suckling behavior of humpback whales and their validity in our datasets was checked to ensure that no suckling events were removed partially or entirely following the segmentation. The pie charts represent the class distribution (suckling versus non-suckling). N = 18, 331 and N = 7,827 before and after segmentation respectively. [file peerj-10-12945-s001.pdf]

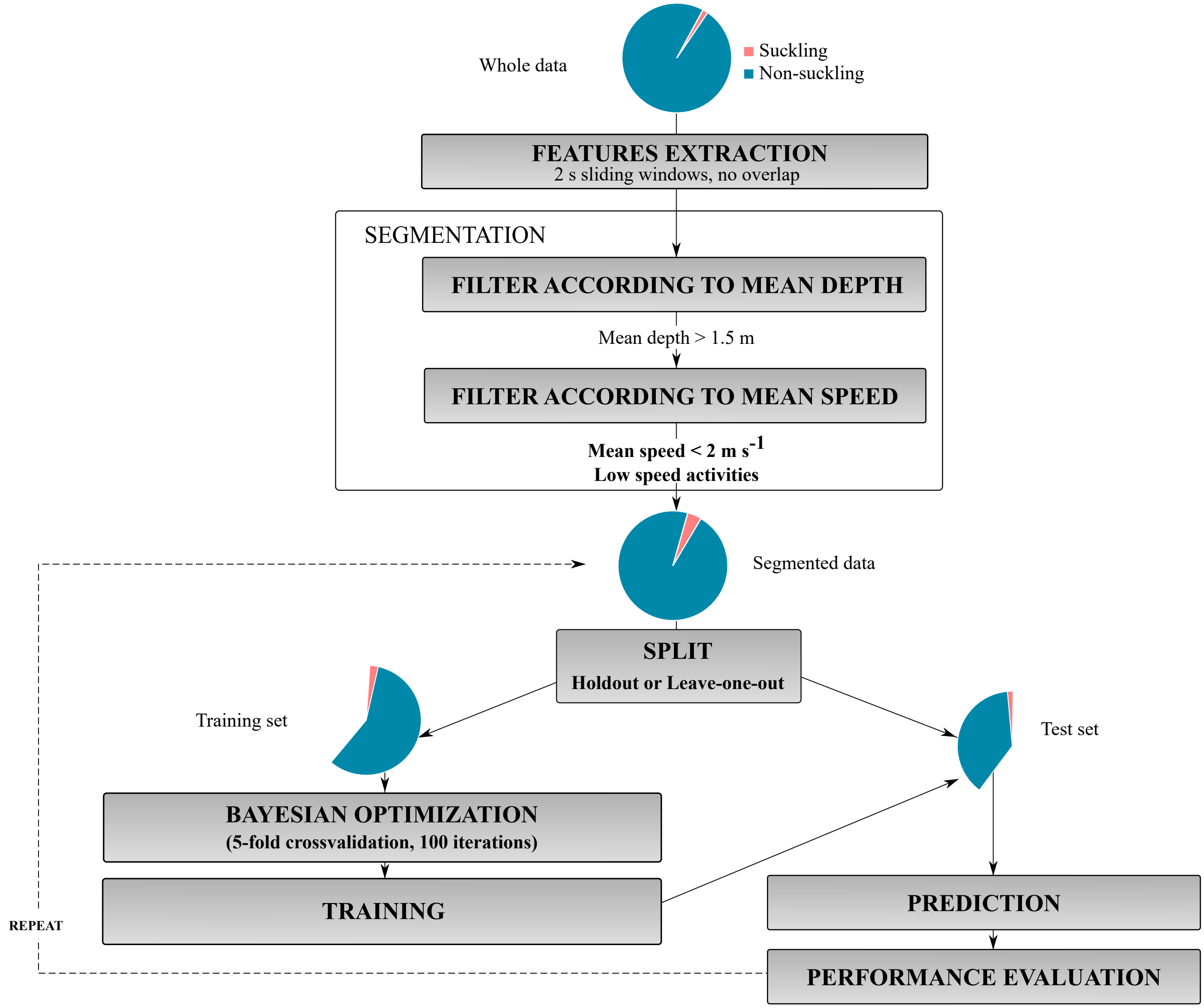
Whole data
Suckling
Non-suckling
FEATURES EXTRACTION
2 s sliding windows, no overlap
SEGMENTATION
FILTER ACCORDING TO MEAN DEPTH
Mean depth > 1.5 m
FILTER ACCORDING TO MEAN SPEED
Mean speed < 2 m s-1
Low speed activities
Segmented data
SPLIT
Holdout or Leave-one-out
Training set
Test set
BAYESIAN OPTIMIZATION
(5-fold crossvalidation, 100 iterations)
TRAINING
PREDICTION
PERFORMANCE EVALUATION
REPEAT
