# Supplementary figures and images for: Characterizing the suckling behavior by video and 3D-accelerometry in humpback whale calves on a breeding ground
Source: PeerJ. 2022 Feb 17;10:e12945. doi: 10.7717/peerj.12945 (PMC8858581; doi:10.7717/peerj.12945)

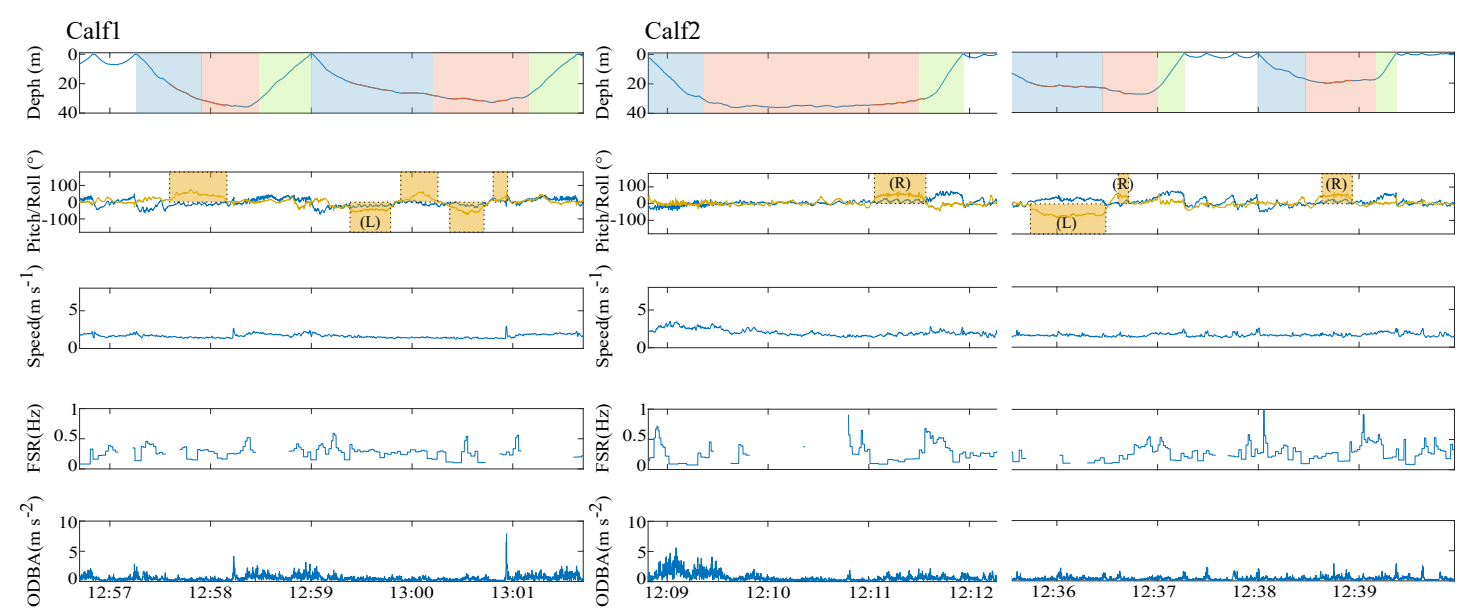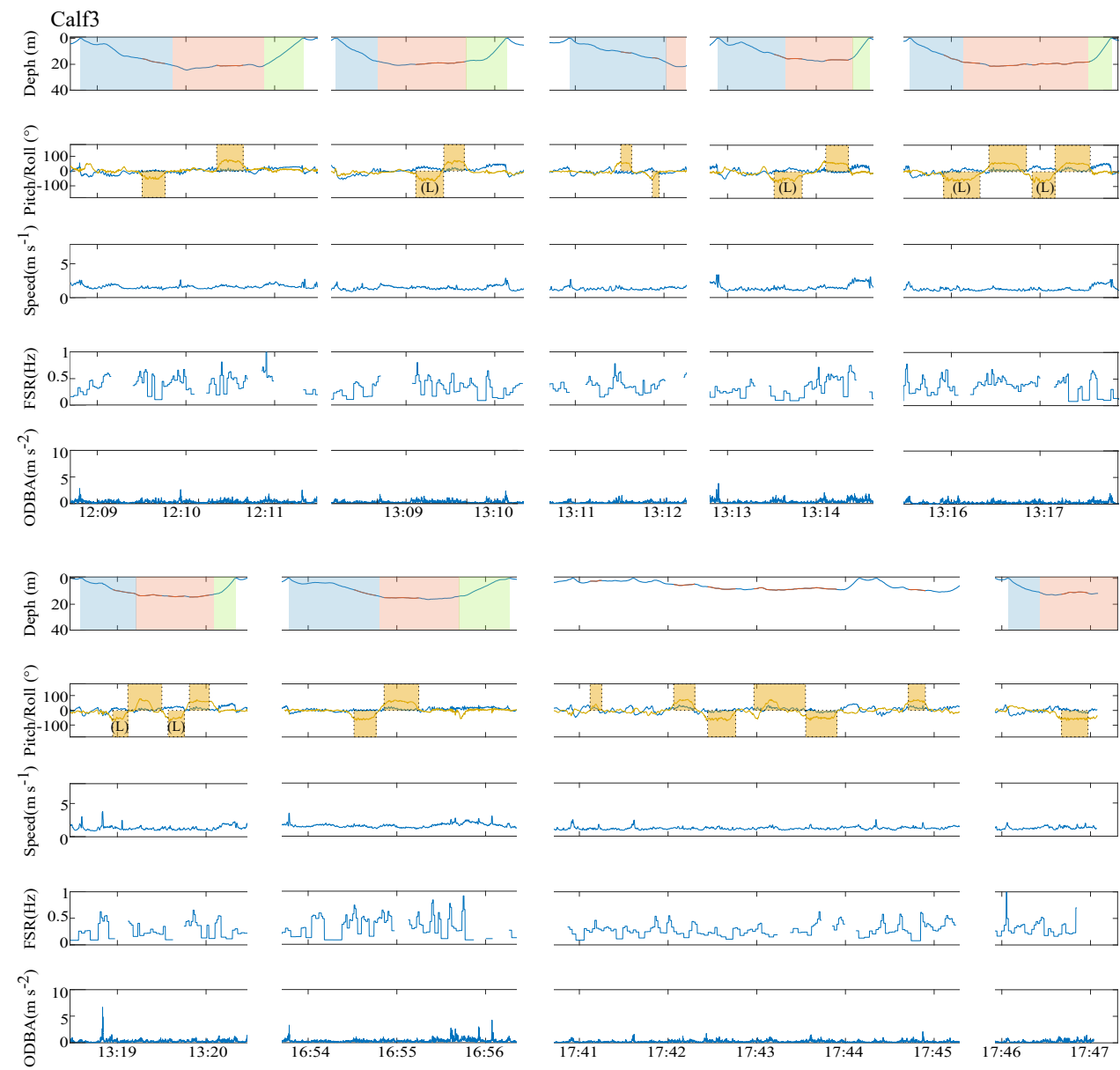

Supplement: Supplemental Information 2 — Blue, red, and green areas in the depth profile correspond to the descent, bottom, and ascent phases of dive respectively. Uncolored areas in the depth profile correspond to surface phases. Suckling events are identified in red in the depth profile and by yellow box in the raw pitch(blue)/roll(yellow) profiles. Yellow box placed on top indicate that the calf was observed rolling to the right side on the corresponding video. Yellow box placed on the bottom indicate that the calf was rather observed rolling to the left side. Events during which the teat suckled by the calf was clearly identified on the corresponding video are marked with (R) or (L): (R) for right teat and (L) for left teat. [file peerj-10-12945-s002.pdf]

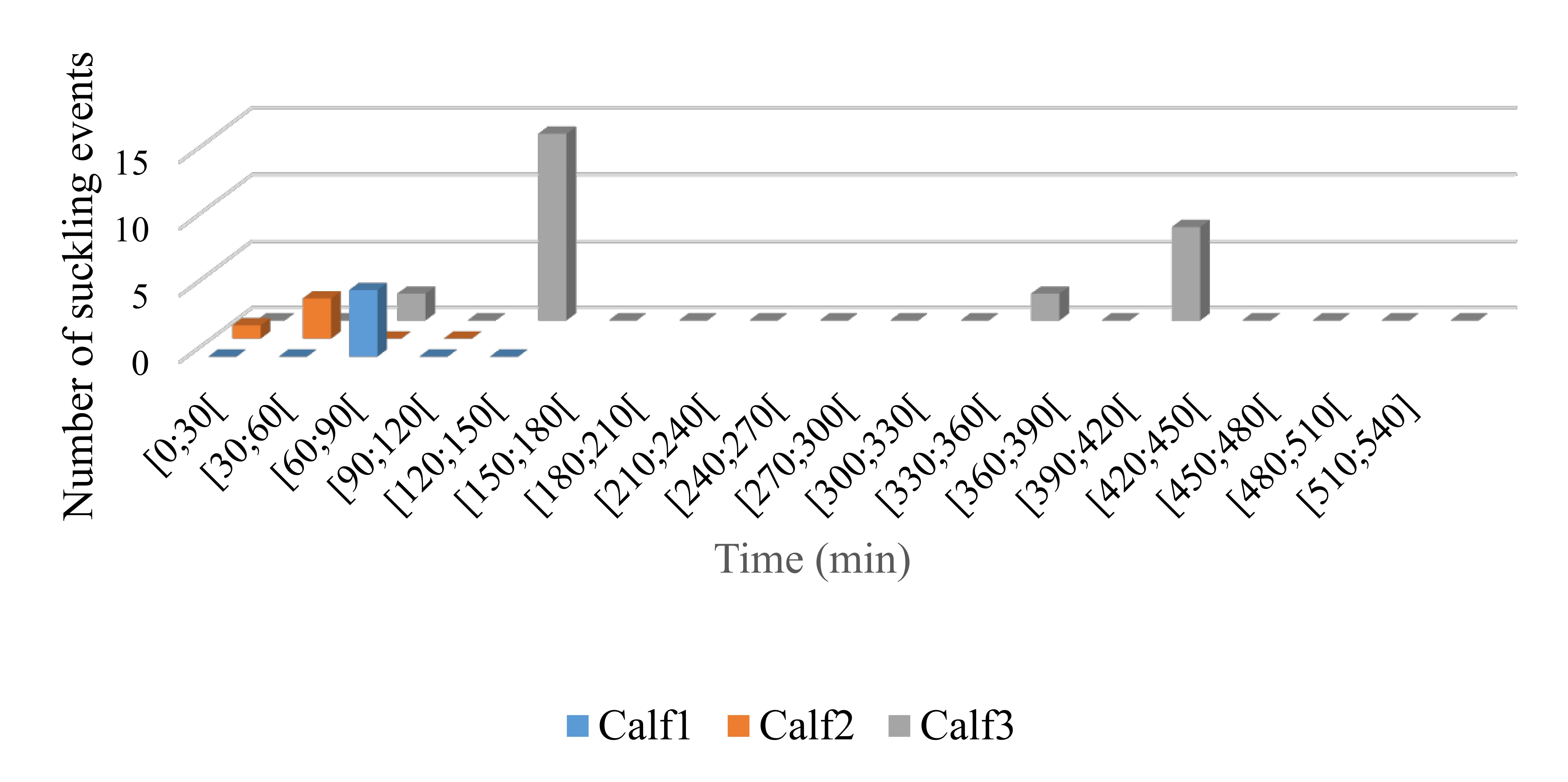

Supplement: Supplemental Information 3 — Blue, red, and grey bars correspond to Calf1, Calf2, and Calf3 respectively. Tag detached at 164 min for Calf1 and at 98 min for Calf2. For Calf3, the tag detached at 521 min. However, only the first 479 min (92%) of the data has been analyzed due to lack of visibility on the video recording as the evening approached. There was no evidence of any trends with suckling frequency increasing later in the deployments. The periods towards the end of the deployments were not necessarily associated with more frequent suckling events. [file peerj-10-12945-s003.jpg]
