# Supplementary material for: Characterizing the suckling behavior by video and 3D-accelerometry in humpback whale calves on a breeding ground
Source: PeerJ. 2022 Feb 17;10:e12945. doi: 10.7717/peerj.12945 (PMC8858581; doi:10.7717/peerj.12945)
Supplement: Supplemental Information 4 [file peerj-10-12945-s004.docx]

|  | **Number** | | | |
| --- | --- | --- | --- | --- |
| **Individuals** | **Non-suckling dives** | | **Suckling dives** | **Total dives** |
|  | **Calf observed staying in close proximity beneath the mother at least 5 s during the dive** | **Calf never observed staying in close proximity beneath the mother at least 5 s during the dive** |  |  |
| Calf1 | 17 | 18 | 2 | 37 |
| Calf2 | 5 | 2 | 3 | 10 |
| Calf3 | 20 | 21 | 9 | 50 |
