# Supplementary material for: Characterizing the suckling behavior by video and 3D-accelerometry in humpback whale calves on a breeding ground
Source: PeerJ. 2022 Feb 17;10:e12945. doi: 10.7717/peerj.12945 (PMC8858581; doi:10.7717/peerj.12945)
Supplement: Supplemental Information 5 — The models included the suckling status and activity phase (descent, bottom, ascent or surface) as fixed effect and individuals as random effect (reference level = bottom and non-suckling, i.e., bottom non-suckling). The data used in the analysis consisted of individual suckling events (18.8 s average duration) and random assortments of non-suckling segments (20 s duration). Significant P (<0.05 in this study) are marked in bold. CI: Confidence interval. SE: Standard Error of estimate. [file peerj-10-12945-s005.docx]

| **Response** | **Fixed effect** | **Effect estimate (β)** | **95% CI** | **SE** | ***t*(96)** | ***P*** |
| --- | --- | --- | --- | --- | --- | --- |
| Average depth rate | (Intercept) | 0.06 | [-0.05, 0.17] | -0.45 | 1.07 | 0.285 |
|  | Suckling | -0.01 | [-0.15, 0.13] | -0.04 | -0.19 | 0.850 |
|  | Descent | 0.60 | [0.48, 0.72] | 1.62 | 9.54 | **< 0.001** |
|  | Surface | -0.02 | [-0.2, 0.17] | -0.05 | -0.19 | 0.849 |
|  | Suckling*Descent | -0.44 | [-0.67, -0.22] | -1.20 | -3.82 | **< 0.001** |
|  | Suckling*Surface | 0.03 | [-0.27, 0.32] | 0.07 | 0.17 | 0.863 |
| Average speed | (Intercept) | 1.62 | [1.28, 1.97] | 0.05 | 9.23 | < 0.001 |
|  | Suckling | -0.10 | [-0.3, 0.11] | -0.21 | -0.91 | 0.363 |
|  | Descent | 0.22 | [0.04, 0.4] | 0.49 | 2.42 | **0.016** |
|  | Surface | 0.30 | [0.02, 0.57] | 0.66 | 2.13 | **0.033** |
|  | Suckling*Descent | -0.21 | [-0.54, 0.12] | -0.46 | -1.23 | 0.220 |
|  | Suckling*Surface | -0.38 | [-0.82, 0.05] | -0.85 | -1.74 | 0.082 |
| Average FSR | (Intercept) | 0.09 | [0.05, 0.13] | -0.63 | 4.22 | **< 0.001** |
|  | Suckling | 0.16 | [0.09, 0.22] | 1.27 | 4.95 | **< 0.001** |
|  | Descent | 0.06 | [0, 0.11] | 0.46 | 2.06 | **0.040** |
|  | Surface | 0.10 | [0.02, 0.18] | 0.79 | 2.41 | **0.016** |
|  | Suckling*Descent | -0.06 | [-0.16, 0.04] | -0.47 | -1.13 | 0.258 |
|  | Suckling*Surface | -0.12 | [-0.25, 0.01] | -0.97 | -1.80 | 0.073 |
| Average ODBA | (Intercept) | 0.43 | [0.18, 0.67] | -0.04 | 3.40 | 0.001 |
|  | Suckling | 0.05 | [-0.11, 0.22] | 0.17 | 0.64 | 0.522 |
|  | Descent | 0.16 | [0.02, 0.3] | 0.49 | 2.19 | **0.028** |
|  | Surface | 0.10 | [-0.12, 0.31] | 0.30 | 0.88 | 0.377 |
|  | Suckling*Descent | -0.20 | [-0.46, 0.06] | -0.62 | -1.51 | 0.132 |
|  | Suckling*Surface | -0.10 | [-0.44, 0.24] | -0.32 | -0.60 | 0.551 |
| Average pitch | (Intercept) | 0.57 | [-8.51, 9.66] | 0.25 | 0.12 | 0.901 |
|  | Suckling | 6.36 | [0.18, 12.53] | 0.42 | 2.02 | **0.044** |
|  | Descent | -18.26 | [-23.58, -12.94] | -1.21 | -6.73 | **< 0.001** |
|  | Surface | -5.05 | [-13.15, 3.06] | -0.34 | -1.22 | 0.222 |
|  | Suckling*Descent | 12.77 | [2.93, 22.61] | 0.85 | 2.54 | **0.011** |
|  | Suckling*Surface | 1.41 | [-11.45, 14.28] | 0.09 | 0.22 | 0.829 |
| \|Average roll\| | (Intercept) | 8.98 | [4.22, 13.74] | -0.58 | 3.70 | **< 0.001** |
|  | Suckling | 38.46 | [31.79, 45.13] | 1.83 | 11.30 | **< 0.001** |
|  | Descent | -0.44 | [-6.29, 5.41] | -0.02 | -0.15 | 0.882 |
|  | Surface | 0.64 | [-7.98, 9.25] | 0.03 | 0.14 | 0.885 |
|  | Suckling*Descent | -0.25 | [-11.06, 10.57] | -0.01 | -0.04 | 0.965 |
|  | Suckling*Surface | -11.56 | [-25.65, 2.52] | -0.55 | -1.61 | 0.108 |
| Average roll rate | (Intercept) | 3.00 | [2.06, 3.94] | -0.34 | 6.25 | **< 0.001** |
|  | Suckling | 1.17 | [-0.05, 2.39] | 0.52 | 1.88 | 0.060 |
|  | Descent | 0.32 | [-0.75, 1.38] | 0.14 | 0.58 | 0.559 |
|  | Surface | 0.28 | [-1.3, 1.87] | 0.13 | 0.35 | 0.726 |
|  | Suckling*Descent | 0.40 | [-1.57, 2.37] | 0.18 | 0.40 | 0.692 |
|  | Suckling*Surface | 1.51 | [-1.06, 4.08] | 0.67 | 1.15 | 0.249 |
