# Supplementary material for: Characterizing the suckling behavior by video and 3D-accelerometry in humpback whale calves on a breeding ground
Source: PeerJ. 2022 Feb 17;10:e12945. doi: 10.7717/peerj.12945 (PMC8858581; doi:10.7717/peerj.12945)
Supplement: Supplemental Information 6 — Comparisons were computed using the R package emmeans. Significant P (<0.05 in this study) are marked in bold. SE: Standard Error of estimate. [file peerj-10-12945-s006.docx]

| **Responses** | **Contrast** | **Estimate (β)** | **SE** | **df** | ***t*** | ***P*** |
| --- | --- | --- | --- | --- | --- | --- |
| Average depth rate | Descent suckling - Descent non-suckling | -0.46 | 0.1 | 98 | 4.8 | **< 0.001** |
|  | Bottom suckling - Bottom non-suckling | -0.01 | 0.07 | 98 | 0.18 | 1 |
|  | Surface suckling - Surface non-suckling | 0.01 | 0.13 | 96 | -0.09 | 1 |
| Average speed | Descent suckling - Descent non-suckling | -0.30 | 0.14 | 96 | 2.21 | 0.243 |
|  | Bottom suckling - Bottom non-suckling | -0.10 | 0.11 | 97 | 0.91 | 0.944 |
|  | Surface suckling - Surface non-suckling | -0.48 | 0.19 | 96 | 2.48 | 0.141 |
| Average FSR | Descent suckling - Descent non-suckling | 0.10 | 0.04 | 98 | -2.32 | 0.197 |
|  | Bottom suckling - Bottom non-suckling | 0.16 | 0.03 | 94 | -4.7 | **< 0.001** |
|  | Surface suckling - Surface non-suckling | 0.04 | 0.06 | 96 | -0.61 | 0.99 |
| Average ODBA | Descent suckling - Descent non-suckling | -0.15 | 0.11 | 96 | 1.36 | 0.752 |
|  | Bottom suckling - Bottom non-suckling | 0.05 | 0.08 | 97 | -0.64 | 0.988 |
|  | Surface suckling - Surface non-suckling | -0.05 | 0.15 | 96 | 0.33 | 0.999 |
| Average pitch | Descent suckling - Descent non-suckling | 19.13 | 4.08 | 96 | -4.68 | **< 0.001** |
|  | Bottom suckling - Bottom non-suckling | 6.36 | 3.17 | 97 | -2.01 | 0.345 |
|  | Surface suckling - Surface non-suckling | 7.77 | 5.76 | 96 | -1.35 | 0.756 |
| Average absolute roll | Descent suckling - Descent non-suckling | 38.21 | 4.54 | 98 | -8.42 | **< 0.001** |
|  | Bottom suckling - Bottom non-suckling | 38.46 | 3.56 | 96 | -10.82 | **< 0.001** |
|  | Surface suckling - Surface non-suckling | 26.89 | 6.33 | 96 | -4.25 | **0.001** |
| Average roll rate | Descent suckling - Descent non-suckling | 1.57 | 0.82 | 98 | -1.9 | 0.406 |
|  | Bottom suckling - Bottom non-suckling | 1.17 | 0.64 | 97 | -1.82 | 0.458 |
|  | Surface suckling - Surface non-suckling | 2.68 | 1.15 | 96 | -2.32 | 0.194 |
