# Supplementary material for: Characterizing the suckling behavior by video and 3D-accelerometry in humpback whale calves on a breeding ground
Source: PeerJ. 2022 Feb 17;10:e12945. doi: 10.7717/peerj.12945 (PMC8858581; doi:10.7717/peerj.12945)
Supplement: Supplemental Information 7 — A Bayesian optimization procedure was run by type of model (Ensemble, KNN, Decision tree, SVM) 30 times in order to select the best model (by seeking to minimize classification error). Values are presented following the format mean ± SD. n represents how often each model was selected by the optimization procedure for each type of classifier over the 30 runs. FPR: False positive rate. [file peerj-10-12945-s007.docx]

| **Classifier Type** | **Models** | ***n*** | **Sensitivity** | **FPR (%)** | **Precision** | **Specificity** | ***F*-score** | **Accuracy (%)** |
| --- | --- | --- | --- | --- | --- | --- | --- | --- |
| **Ensemble** | AdaBoostM1 | 17 | 0.71±0.04 | 0.38±0.09 | 0.89±0.02 | 1 | 0.79±0.02 | 98.48±0.13 |
| (30 runs) | Bag (Random forest) | 4 | 0.58±0.05 | 0.27±0.1 | 0.9±0.04 | 1 | 0.71±0.04 | 98.08±0.26 |
|  | GentleBoost | 9 | 0.64±0.14 | 0.46±0.27 | 0.84±0.12 | 1 | 0.72±0.13 | 98.11±0.75 |
|  |  |  |  |  |  |  |  |  |
| **K‐Nearest Neighbors (KNN)** | Cityblock | 15 | 0.66±0.05 | 0.51±0.24 | 0.85±0.05 | 0.99 | 0.74±0.03 | 98.14±0.21 |
| (30 runs) | Cosine | 1 | 0.6 | 0.47 | 0.84 | 1 | 0.7 | 97.99 |
|  | Euclidean | 2 | 0.55±0.06 | 0.63±0.28 | 0.79±0.06 | 0.99 | 0.65±0.02 | 97.6±0.05 |
|  | Minkowski | 12 | 0.69±0.05 | 0.44±0.12 | 0.87±0.03 | 1 | 0.76±0.03 | 98.32±0.21 |
|  |  |  |  |  |  |  |  |  |
| **Decision tree:** | Deviance | 19 | 0.54±0.11 | 0.99±0.51 | 0.71±0.08 | 0.99±0.01 | 0.6±0.06 | 97.21±0.29 |
| (30 runs) | Gdi | 11 | 0.54±0.05 | 0.84±0.37 | 0.74±0.06 | 0.99 | 0.62±0.03 | 97.36±0.22 |
|  |  |  |  |  |  |  |  |  |
| **Support Vector Machine (SVM)** | Gaussian | 26 | 0.58±0.21 | 0.55±0.32 | 0.82±0.07 | 0.99 | 0.64±0.23 | 97.78±0.7 |
| (30 runs) | Linear | 2 | 0.52±0.01 | 0.72±0.16 | 0.75±0.04 | 0.99 | 0.61±0.01 | 97.41±0.14 |
|  | Polynomial | 2 | 0.66±0.02 | 1.76±0.56 | 0.61±0.07 | 0.98±0.01 | 0.63±0.03 | 96.93±0.45 |
